# Selective inhibition of soluble TNF using XPro1595 relieves pain and attenuates cerulein-induced pathology in mice

**DOI:** 10.1186/s12876-021-01827-0

**Published:** 2021-05-28

**Authors:** Rajasa Randhi, Melissa Damon, Kirsty J. Dixon

**Affiliations:** Department of Surgery, Virginia Commonwealth University, 1101 E. Marshall St, Richmond, VA 23298 USA

**Keywords:** Acute pancreatitis, Cerulein, Mice, Inflammation, Cytokines, TNF, TNFR1, Neuropathic pain

## Abstract

**Background:**

Symptoms associated with acute pancreatitis can be debilitating, and treatment remains a challenge. This study aimed to investigate the efficacy of selectively inhibiting the soluble form of TNF (solTNF) using the biologic XPro1595 in a mouse model of acute pancreatitis.

**Methods:**

Acute pancreatitis was induced in adult male C57Bl/6J mice by administering cerulein (8 injections of 50 µg/kg I.P., spaced an hour apart), with XPro1595 (10 mg/kg, S.C.) or vehicle being administered approximately 18 h after the last injection. Serum was collected 6 or 18 h after the last cerulein injection, pancreatic tissue was collected 2 and 7 days post-induction, and brain hippocampal tissue was collected at 7 days post-induction. The animal’s pain level was assessed 3, 5 and 7 days post-induction.

**Results:**

The induction of acute pancreatitis promoted a strong increase in serum amylase levels, which had receded back to baseline levels by the next morning. XPro1595 treatment began after amylase levels had subsided at 18 h, and prevented pancreatic immune cell infiltration, that subsequently prevented tissue disruption and acinar cell death. These improvements in pathology were associated with a significant reduction in mechanical hypersensitivity (neuropathic pain). XPro1595 treatment also prevented an increase in hippocampal astrocyte reactivity, that may be associated with the prevention of neuropathic pain in this mouse model.

**Conclusion:**

Overall, we observed that selectively inhibiting solTNF using XPro1595 improved the pathophysiological and neurological sequelae of cerulein-induced pancreatitis in mice, which provides support of its use in patients with pancreatitis.

## Introduction

Pancreatitis is a leading cause for gastrointestinal disease-related hospital admissions, with symptoms including moderate to severe upper abdominal epigastric pain, nausea and vomiting [1]. Studies in patients and animals have identified the disease course and severity of pancreatitis is mostly governed by inflammatory cells that drive the upregulation of local and systemic immune responses, of which a major contributor is the inflammatory cytokine tumor necrosis factor (TNF). It has long been known that TNF production promotes the induction of inflammatory genes, recruitment of immune cells and acinar cell death [2–5], and this prompted investigation of traditional TNF inhibitory therapies to prevent or reduce these pathologies and associated symptoms. Early studies in rodents modulating TNF ligand and receptor activity used TNF receptor fusion proteins or anti-TNF antibodies (e.g., Etanercept, Infliximab and Adalimumab) showed promise with reduced pancreatic pathologies such as edema, inflammation, necrosis and vacuolization [6, 7], with similar positive outcomes seen in patients when TNF inhibitors were administered to treat other cooccurring conditions [8, 9]. Unfortunately, the abundance of side-effects in these traditional TNF inhibitors (including immunological dysfunction and even the induction of pancreatitis itself [10–13]), combined with their apparent inability to reduce mortality in sepsis patients [14, 15] dampened enthusiasm for their further use in patients with pancreatitis. More than 2 decades on however, additional meta-analysis’ of data in sepsis patients revealed an overall improvement in survival rates [16], when studies are sufficiently powered, which likely prompted a re-examination of these traditional TNF inhibitors in patients with pancreatitis [17, 18]. None-the-less, these traditional inhibitors still promote severe side-effects, and their use should be cautioned in patients.

The abundance of side-effects with traditional TNF inhibitors is likely due to the differences in TNF receptor subtype functions, that have complicated the TNF field until recently. TNF is first produced as a transmembrane protein (tmTNF) that preferentially activates TNF receptor 2 (TNFR2: CD120b or p75/p80) [19], but once cleaved from the cell membrane it exists in a soluble form (solTNF) and preferentially activates TNF receptor 1 (TNFR1: CD120a or p55/p60) [19]. Although both TNFR1 and TNFR2 can trigger some common signaling pathways [20], TNFR2 activation generally promotes beneficial outcomes such as cell survival, induction of neurogenesis, and promotion of CNS autoimmunity [21, 22], while TNFR1 activity generally promotes detrimental outcomes such as cell death, aberrant neuronal plasticity, and exacerbation of the existing inflammatory response [21, 23, 24]. Indeed, within the pancreas TNFR1 activity is known to exacerbate cell death, and promote inflammation and edema [25], while TNFR2 promotes pancreatic regeneration [26, 27]. Unfortunately, traditional TNF inhibitors are unable to distinguish between the different TNF ligand or receptor subtypes. Therefore, being able to selectively block the activity of solTNF/TNFR1, while sparing the activity of tmTNF/TNFR2 activity, would likely prove beneficial to patients with pancreatitis. For this reason, a novel ‘second generation’ TNF inhibitor was developed that selectively inhibits only the soluble form of TNF (solTNF: XPro1595). XPro1595 has been successfully used in numerous pre-clinical inflammatory disease models with no known side-effects [28–32]. In recent clinical trials in cancer patients it has been shown to be safe and well tolerated [33], with a second trial in Alzheimer Disease patients showing an ability to reverse neuroinflammation within the brain [34]. Therefore, we sought to investigate whether using XPro1595 to selectively bind and neutralize solTNF can improve outcomes in a mouse model of pancreatitis.

## Methods

### Animals

Male C57Bl/6J mice aged 2 to 4 months were used for the current study. Animals were housed with a maximum of 5 mice per cage, in standard cages, in a 12-h light/dark cycle, with food and water ad libitum. Procedures related to animal use were approved and carried out in accordance with the Virginia Commonwealth University Institutional Animal Care and Use Committee (protocol AD10001704) in accordance with NIH care and use of laboratory animals). This study was also carried out in accordance with the ARRIVE guidelines.

### Study design

The study groups included mice treated with (C) or without (NC) cerulein, that survived 6 h, 18 h, 2 days or 7 days after the last cerulein injection, and either treated with XPro1595 (XP) or vehicle (V). Number of animals in each group are as follows: 6 h survival NC = 3, C = 3; 18 h NC = 3, C = 3; 2 days NC + V = 5, C + V = 3, C + XP = 3; 7 days NC + V = 4, NC + XP = 3, C + V = 4, C + XP = 4. Serum was collected from animals surviving to 6 and 18 h, while animals surviving for 2 days had their pancreas collected, and animals surviving for 7 days underwent neurological testing on days 3, 5 and 7, prior to having their pancreas and brain collected for analysis (on day 7).

### Acute pancreatitis model

On a single day, mice received 8 intraperitoneal injections of cerulein (50 µg/kg; Sigma) or vehicle (0.9% saline), over 7 h, spaced an hour apart each. Injections were alternated between the left and right sides of the abdomen to minimize any possible irritation from multiple needle injuries. The next morning (approximately 18 h after the last cerulein injection) mice were either sacrificed, or received a subcutaneous injection of either XPro1595 (10 mg/kg, INmuneBio) or vehicle (0.1 M PBS) for survival to 2 or 7 days.

### Blood collection and amylase analysis

Increased expression of serum amylase activity in patients supports the diagnosis of acute pancreatitis, therefore mice were anaesthetized with ketamine (75 mg/kg) and xylazine (14 mg/kg) by intraperitoneal injection, cardiac blood was collected into 1.5 ml polypropylene tubes and incubated on ice for 15 min, prior to centrifugation at 2000 rpm for 10 min. The resultant supernatant was removed and stored at -80 °C. On the day of analysis, serum was defrosted on ice, and approximately 40 µl loaded into a VETSCAN ‘Comprehensive Diagnostic’ rotor (Abaxis Inc.), prior to being analyzed in a Vetscan VS2 (Abaxis Inc.).

### Histological Preparation, Staining and Immunohistochemistry

Mice were anaesthetized with ketamine (75 mg/kg) and xylazine (14 mg/kg) by intraperitoneal injection, prior to undergoing transcardial perfusion using approximately 15 ml PBS, followed by approximately 4% paraformaldehyde (Sigma). Tissue was dissected, stored in 4% paraformaldehyde for 2 h, cryoprotected in 20% sucrose in PBS for 48 h, and then quickly frozen in OCT over isopentane on dry ice, and stored at − 80 °C. Serial frozen coronal sections were cut 40 µm thick through the pancreas and hippocampus. Some slides underwent hematoxylin and eosin (H&E) staining to assess pancreas inflammatory infiltrates and tissue integrity. Other slides underwent immunohistochemical analysis to further assess inflammatory state. Slides containing sections of either pancreas or brain were permeabilized with 0.2% triton X-100 (Sigma) in 2% fish gel in PBS solution and immunohistochemically labelled with the primary antibody (1:2000 rabbit anti-GFAP, Dako; 1:2000 rabbit anti-IBA-1, Wako) overnight at 4 °C. Sections were washed 3 times in PBS, incubated in fluorescent secondary antibodies (1:500, Molecular Probes) for 30 min at room temperature, washed an additional 3 times in PBS, and coverslipped in Prolong Gold Antifade mounting medium containing DAPI (ThermoFisher). Sections were photographed (pancreas at 10× and 40×; brain at 40×s) with equal exposure on an Olympus CK-2 inverted microscope, connected to a 3MP Amscope digital camera (MU300-CK) with Amscope Software version 3.2, prior to analysis using NIH ImageJ version 1.52a.

### Histological analysis

To assess pancreatic pathology, photographs of H&E stained pancreatic sections were semi-quantitated for immune cell infiltration, tissue integrity, acinar cell atrophy and intralobular duct integrity on an arbitrary scale of 0 to 2, within each field of view [35–38]. Pancreatic immune cell infiltration was assessed at 2 and 7 days post-induction where a score of 0 = no inflammatory cells present; 1 = some inflammatory cells present; 2 = many inflammatory cells present. At 7 days we also semi-quantitated pancreatic tissue integrity (spaces between acinar cell clusters: 0 = normal pathology; 1 = some spaces evident; 2 = large amount of space evident appearing similar to a ‘cracking’ effect), acinar cell atrophy (0 = normal pathology; 1 = acinar atrophy present but not immediately apparent; 2 = acinar atrophy prevalent throughout the tissue), and integrity of intralobular duct (degree of invasion of spaces normally occupied by vessels within the large pancreatic lobules: 0 = normal pathology, 1 = some invasion present but not immediately apparent, 2 = large amount of invasion present). Values for each photograph were averaged per section, per animal, and then per group.

To assess the extent of circulating macrophage infiltration within the pancreas, the immunohistochemistry images were quantitated for the level of IBA-1 expression [39, 40]. Images were imported into ImageJ software (NIH), converted to gray scale, thresholded, and the area fraction of pixels positive for IBA-1 was quantitated, the values for each photograph were averaged per section, per animal, and then per group [41, 42]. The same protocol was applied to brain sections containing hippocampus at 7 days post-induction to quantitate astrocyte reactivity using the GFAP antibody.

### Mechanical von Frey Hindpaw neuropathic pain

Many pre-clinical rodent models of acute pancreatitis display both pancreatic and referred neuropathic pain in both the abdomen and hindpaws [43–45]. To assess the role of solTNF in the induction of pancreatitis-induced neuropathic pain, we measured the level of hindpaw mechanical hypersensitivity at 3, 5 and 7 days post-induction. In a dimly lit room, a 10″ × 19″ extension window screen (Thermwell) was fully extended and placed atop 2 polystyrene boxes, with a desk lamp placed behind and just under the height of the screen, angled towards the investigator. Four mice at a time were placed on top of the screen, with a 600 ml glass beaker (Pyrex) placed over the top of each mouse to prevent escape. A disposable underpad was draped over the beakers to minimize any light and/or movement stimulation. After 15 min acclimatization under the beaker, hindpaw hypersensitivity was assessed by holding the von Frey filament (Bioseb) handle under the screen, and slowly raising the end of the filament up through the screen to press against the under-side of the mouse’s hindpaw walking pad until a slight bend was observed in the fiber. Continued advancement/bending of the filament does not necessarily produce more force of application. The investigator tested the lightest filament first, and sequentially tested up through the filament sizes until a positive result was established. A positive result was the mouse noticing 3 out of 5 consecutive tests for each filament, defined as the mouse withdrawing its foot, licking or shaking its foot, or rapidly moving its body away from the stimulus. Once a positive result was established for each mouse, the testing was concluded for that mouse for that day. The testing occurred as rapidly as possible to reduce restraint distress, although it was noticed that mice would often fall asleep during testing, which required gentle tapping from underneath the screen to wake up the animal.

### Statistical analysis

All data were assessed for homogeneity of variance, after which statistical analysis was performed. Histological differences were assessed using the Student’s t-test, and behavioral differences (intra- and inter-group analysis) were assessed using two-way repeated measures analysis of variance with Student–Newman–Keuls method post hoc in SigmaPlot 13.0 where significance was < 0.05. Data in figures are expressed as mean ± standard error of the mean.

## Results

### Cerulein administration induces a temporary increase in serum amylase levels in mice

**Fig. 1:**
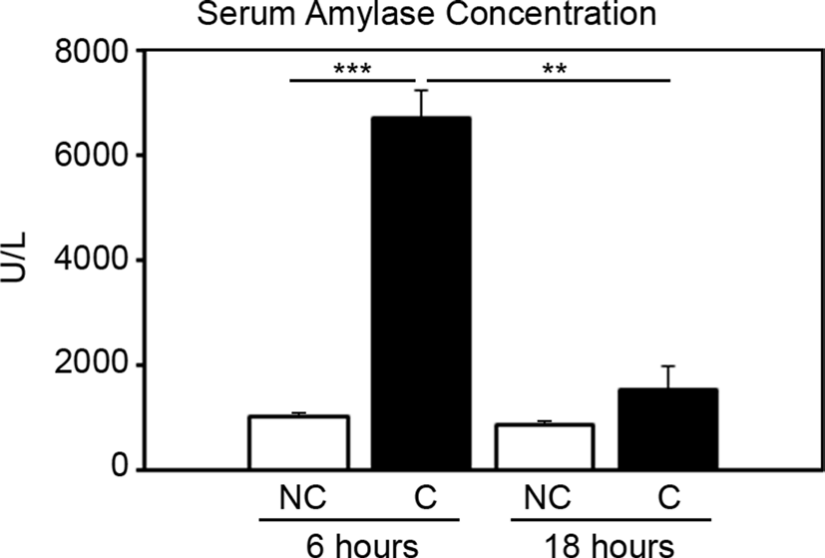
Cerulein administration promotes induction of pancreatitis. Acute pancreatitis was induced by 8 intraperitoneal injections of cerulein (50 µg/kg) or vehicle to mice, spaced an hour apart each, and serum amylase concentrations were measured 6 and 18 h after the last cerulein injection. Cerulein-treated mice displayed a temporary spike in serum amylase concentration at 6 h, that had reduced back to baseline levels by the next morning (n = 3 per group). *NC* non-cerulein, *C* cerulein, **p < 0.01, ***p < 0.001

To confirm whether 8 intraperitoneal injections of cerulein space an hour apart, over the course of 7 h increases serum amylase levels, we collected serum 6 and 18 h after the last cerulein injection. Six hours after the last cerulein injection, a sixfold increase (6687 ± 523 U/L) in serum amylase expression was observed, compared to vehicle-treated mice (1030 ± 22 U/L: Fig. 1), but regressed back to baseline levels 12 h later (1530 ± 448 U/L), and no longer significantly different to vehicle-treated non-pancreatitis mice (863 ± 23 U/L).

### Selective inhibition of solTNF prevents immune cell infiltration into the pancreas following cerulein-induction of acute pancreatitis

**Fig. 2:**
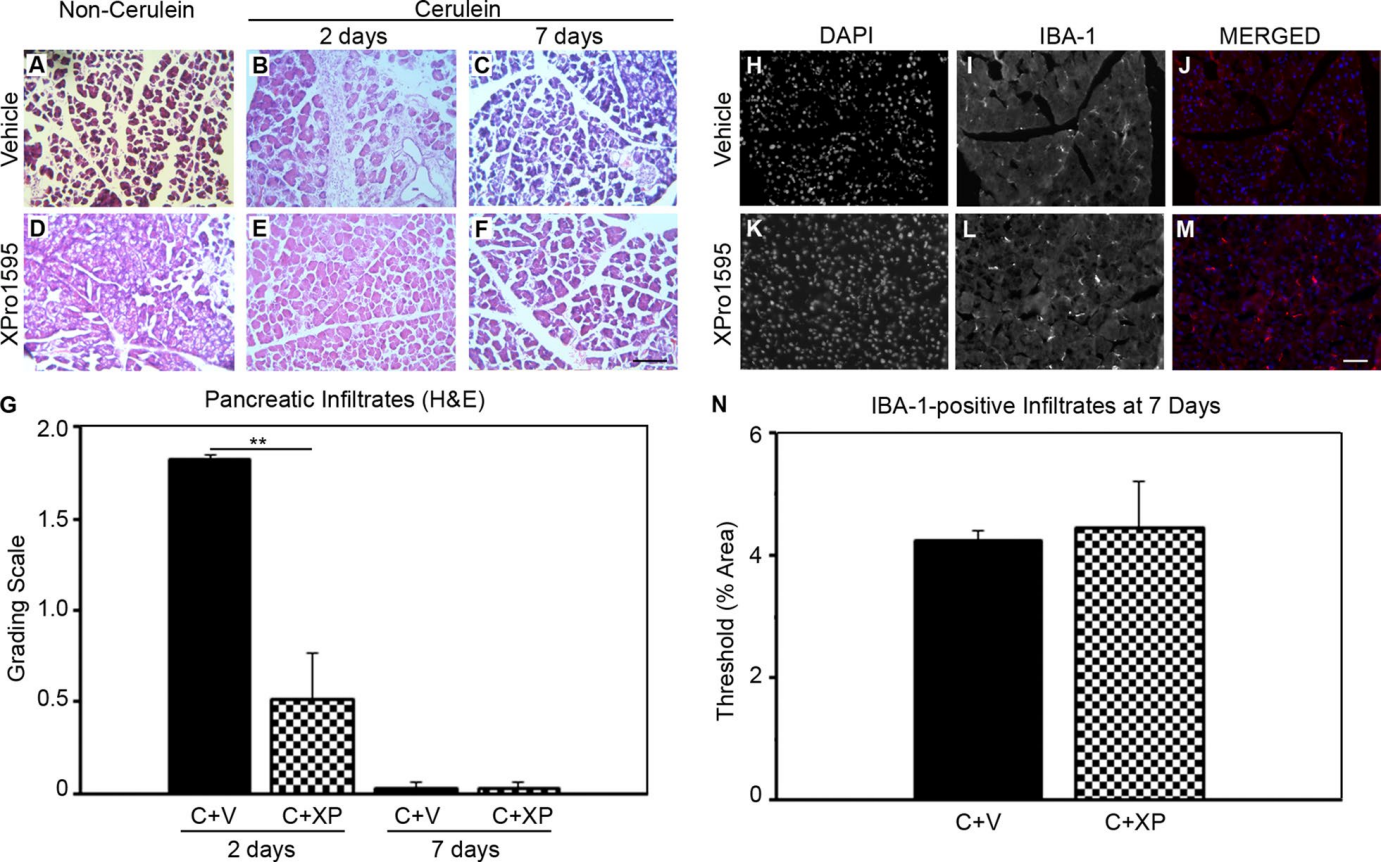
XPro1595 treatment attenuates cerulein-induced pancreatic inflammatory infiltrates. Photomicrographs illustrate hematoxylin and eosin stained sections of mouse pancreas in non-cerulein (**a**, **d**) and cerulein (**b**, **c**, **e**, **f**) mice, 2 days after cerulein/vehicle administration. Vehicle-treated non-cerulein mice had an absence of inflammatory infiltrates (**a**) (n = 5), but cerulein treatment promoted an influx of inflammatory cells within 2 days (**b**) (n = 3), which had resolved by 7 days (**c**, **d**) (n = 4). XPro1595 treatment significantly reduced the immune cell infiltration at 2 days (**e**) (n = 3), that was even further reduced by 7 days (**f**) (n = 4). A week following pancreatitis induction the circulating macrophage population was also minimal in cerulein-treated mice, independent of vehicle or XPro1595 treatment (n = 4 each). *C* cerulein, *V* vehicle, *XP* XPro1595, **p < 0.01, scale bars = 100 µm

Forty eight hours after acute pancreatitis induction, vehicle-treated mice had a significant influx of inflammatory cells, as observed by H&E (score = 1.8 ± 0.03) (Fig. 2a, b, g). In comparison, the XPro1595-treated acute pancreatitis mice had a significant reduction in the number of inflammatory cells present at the same time point (score = 0.5 ± 0.4) (Fig. 2d, e, g). By 7 days post-induction, inflammatory infiltrates were absent in both XPro1595- and vehicle-treated acute pancreatitis groups (score = 0) (Fig. 2c, f, g). To confirm the resolution of an inflammatory response in the same 7-day tissue, we semi-quantitated the level of ionizing binding adaptor protein 1 (IBA-1), as a marker of circulating macrophages within pancreatic islets (Fig. 2h–m). ImageJ quantitation of high-resolution pancreatic images revealed no differences in IBA-1 expression between vehicle- or XPro1595-treated mice (Fig. 2n).

### Selective inhibition of solTNF prevents adverse pancreatic pathology 7 days following cerulein-induction of acute pancreatitis

**Fig. 3:**
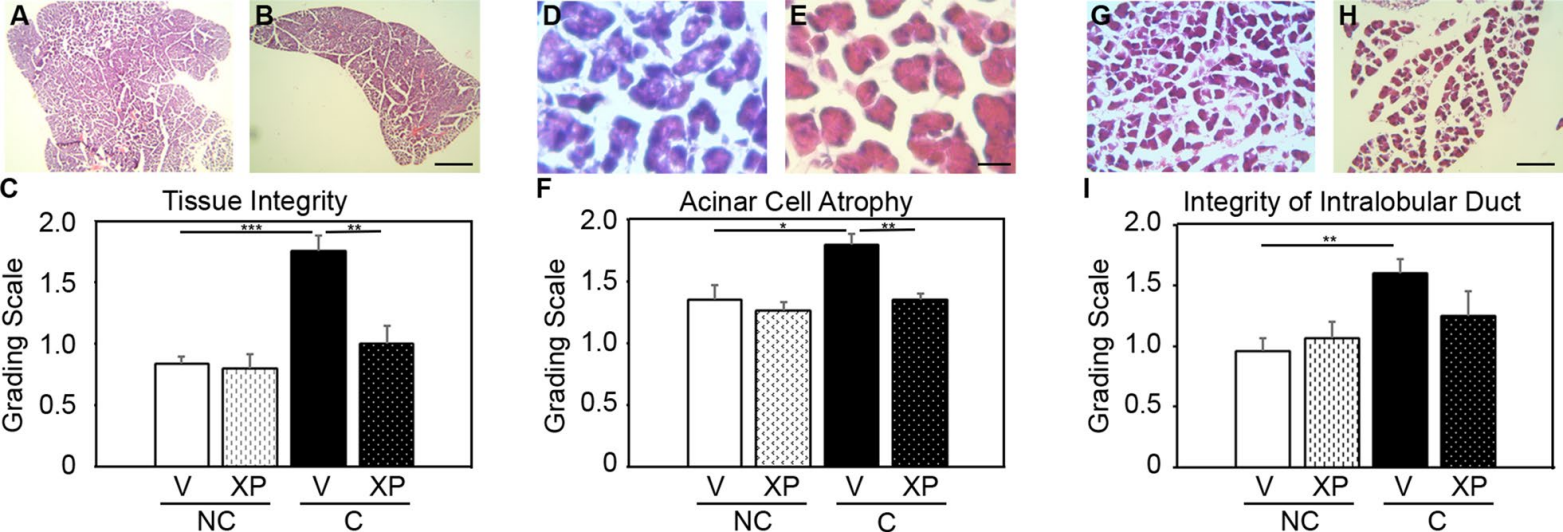
Selective inhibition of solTNF in mice with acute pancreatitis prevents pancreatic tissue degradation at 7 days after induction. Photomicrographs illustrate H&E stained pancreatic sections from mice with acute pancreatitis (cerulein treated) with either vehicle—**a** (n = 4) or XPro1595-treatment **b** (n = 4), where quantification shows vehicle-treated pancreatitis mice have significant deterioration of tissue integrity (**c**), compared to pancreatitis mice treated with XPro1595 (n = 4), or non-pancreatitis mice (n = 3–4). In accordance, the vehicle-treated pancreatitis mice also display an exacerbated level of pancreatic acinar cell atrophy (**d**) that is not observed in the XPro1595 treated pancreatitis group (**e**, **f**). A week after induction of pancreatitis the intralobular ducts in the vehicle-treated group appeared to be invaded by groups of acini spreading apart (**g**), although this was less prominent in the XPro1595-treated group (**h**, **i**). *NC* non-cerulein, *C* cerulein, *V* vehicle, *XP* XPro1595, *p < 0.05, **p < 0.01, ***p < 0.001, scale bar in **a** and **b** = 500 µm, scalebar in **d** and **e** = 25 µm, scalebar in **g** and **h** = 500 µm

To determine whether the early alteration in pancreatic inflammatory infiltrates in XPro1595-treated mice subsequently improved pancreatic pathology, we semi-quantitated pancreatic tissue integrity, acinar cell atrophy, and intralobular duct pathology. We identified that mice with acute pancreatitis had reduced pancreatic tissue integrity (cracking within small cell clusters: NC + V = 0.84 ± 0.05; C + V = 1.75 ± 0.12: Fig. 3a, c), while XPro1595 treatment prevented this effect (C + XP = 1.0 ± 0.14: significantly less than vehicle-treated pancreatitis mice, and not different to non-pancreatitis mice (Fig. 3b, c). We also observed that acute pancreatitis promotes acinar atrophy within 7 days (C + V = 1.8 ± 0.08; Fig. 3d, f), compared to vehicle-treated non-pancreatitis mice (NC + V = 1.35 ± 0.12; Fig. 3f), which is also prevented by XPro1595 treatment (C + XP = 1.35 ± 0.05: significantly less than vehicle-treated pancreatitis mice, and not different to non-pancreatitis mice) (Fig. 3e, f). We further assessed the integrity of the intralobular duct between large pancreatic lobules. Acute pancreatitis promoted significant disruption of pathology between the large pancreatic lobules, whereby acinar clusters within lobules often invaded these spaces (1.6 ± 0.115: Fig. 3g, i). This effect was not observed in the XPro1595-treated pancreatitis group (1.25 ± 0.2: not significantly different to non-pancreatitis mouse group) (Fig. 3h, i).

### Selective inhibition of solTNF using XPro1595 attenuates cerulein-induced neuropathic pain

**Fig. 4:**
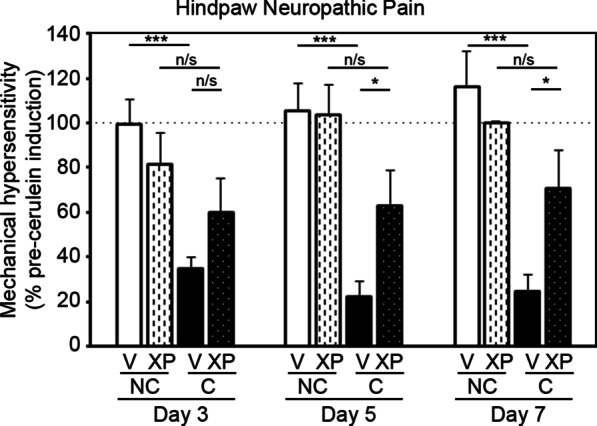
Soluble TNF activity reduces hindpaw mechanical hyper-sensitivity following induction of acute pancreatitis in mice. Graph shows mechanical hypersensitivity of the hindpaw when touched by von Frey filaments, as a percentage of pre-cerulein baseline data for each mouse. No differences were observed in hypersensitivity of non-pancreatitis mice between baseline and on days 3, 5 and 7, independent of treatment (n = 6–8). In contrast, vehicle-treated pancreatitis mice displayed significantly more hypersensitivity than non-pancreatitis control mice at each time point assessed during the first week (n = 9). Treating mice with XPro1595 prevented this hypersensitivity at all time-points tested (n = 9), and this group displayed significantly less hypersensitivity than the vehicle-treated pancreatitis group on the last 2 days of testing. *NC* non-cerulein, *C* cerulein, *V* vehicle, *XP* XPro1595, *p < 0.05, ***p < 0.001

We quantitated the level of hindpaw mechanical hypersensitivity at 3, 5 and 7 days after acute pancreatitis induction in mice treated with and without XPro1595. Vehicle-treated non-pancreatitis mice were assessed to establish baseline sensitivity response, set at 100%. The hindpaw sensitivity of XPro1595-treated non-pancreatitis mice was not different from vehicle-treated non-pancreatitis mice over the testing period (Fig. 4). Next, we identified that vehicle-treated acute pancreatitis mice displayed persistent hindpaw hypersensitivity, beginning from the first day of testing (day 3) until the last (day 7). In contrast, the hindpaw hypersensitivity of XPro1595-treated pancreatitis mice was not significantly different to baseline control mice at any time point tested, and was significantly better than vehicle-treated pancreatitis mice 5 and 7 days later.

### Effect of inhibiting solTNF using XPro1595 on hippocampal astrocyte reactivity after induction of pancreatitis in mice

**Fig. 5:**
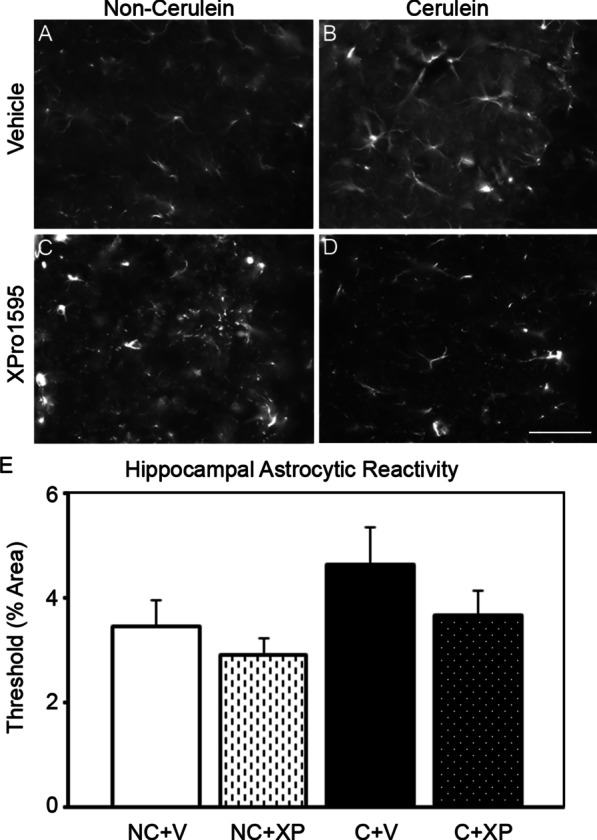
Hippocampal GFAP expression is more pronounced in cerulein-treated mice without XPro1595 administration. CA1 hippocampal GFAP expression was semi-quantitated 7 days following induction of pancreatitis in mice. Non-pancreatitis mouse groups, independent of vehicle or XPro1595 treatment, did not display reactive astrogliosis (**a**, **c**) (n = 3–4). In contrast, the vehicle-treated pancreatitis tissue (**b**) had numerous reactive astrocytes and when quantitated displayed a tendency for increased GFAP expression (n = 4), compared to the non-pancreatitis groups, but was not statistically significantly different (**e**). XPro1595 treated pancreatitis mice prevented the induction of reactive astrocytes (**d**) (n = 4). *NC* non-cerulein, *C* cerulein, *V* vehicle, *XP* XPro1595, scalebar = 100 µm

To assess whether hippocampal inflammation may play a role in pancreatitis-induced mechanical hypersensitivity, we semi-quantitated hippocampal GFAP expression, as a marker of astroglial reactivity 7 days after cerulean administration. We observed that induction of acute pancreatitis increased the tendency for hippocampal CA1 astrocyte reactivity (increased GFAP expression) within 7 days, compared to non-pancreatitis mice (Fig. 5a, b, e), which was not apparent in the XPro1595-treated pancreatitis group (Fig. 5c–e).

## Discussion

### XPro1595 administration during a clinically relevant window

The onset of acute pancreatitis coincides with a spike in the blood pancreatic enzymes amylase and lipase [46], a local and systemic inflammatory response, and is associated with abdominal tenderness and pain, as well as nausea and vomiting. The presence of an amylase spike in the current mouse study suggests similarities exist between the rodent model and patient sequalae, prompting the administration of XPro1595 after the amylase levels return to baseline, i.e. during a clinically relevant window.

### Selective inhibition of soluble TNF prevents acute pancreatitis pathological sequalae

Although the pathogenesis of acute pancreatitis is not fully understood, a number of conditions are known to induce this disorder including pancreatic duct obstruction, alcoholism, and a genetic mutation, which ultimately cause a severe inflammatory response with in the pancreas. Management of this condition includes removal of any obstruction/s, nutritional regulation (including pancreatic enzyme supplementation and hydration), and pain management [47]. Pharmacologic interventions have targeted the inhibition of proteolytic enzymes using broad spectrum anti-protease inhibitors that showed variable outcomes in animals if delivered before disease onset [48, 49]. Unfortunately, these inhibitors failed to show any effect in patients, possibly due to their administration after peak enzymatic activity, but which may be unavoidable given the short peak in enzymatic activity in patients [50, 51]. Another pharmacological direction is to regulate the immune response, which also displays different phases of pro- and anti-inflammation, but which may represent a more clinically relevant timepoint. Animal studies inhibiting pro-inflammatory mediators including IL-6, and ICAM, or bolstering anti-inflammatory mediators such as IL-10 have also shown variable successes [52–56], but enthusiasm for their use has diminished due to limited benefits observed in regulating the inflammatory response [57–60]. Notably, early studies in rodents using TNF inhibitors showed promise with improved pancreatic pathology [6, 7, 25], and with positive outcomes also seen in sepsis patients [8, 9], although their abundance of side-effects, likely due to TNFR2 inhibition, dampened enthusiasm for their further use. Now, a first in its class inhibitor XPro1595, that can selectively neutralize only solTNF, has been shown to reduce disease severity by limiting the initial infiltration of inflammatory cells into the pancreas. This early reduction in inflammatory severity was subsequently associated with improved pancreatic pathology (overall tissue integrity, acinar cell atrophy, and intralobular duct integrity). Future studies will still be required to assess the underlying mechanism of a reduced inflammatory response on each component of the aforementioned improvements in pathology, however it has long been known that pancreatic TNF/TNFR1 activity indeed exacerbates cell death, and promotes inflammation and edema [25].

### Selectively inhibiting soluble TNF prevents pancreatitis-associated pain

Assessing the symptoms of acute pancreatitis in rodents, especially that of visceral pain is challenging. None-the-less, many preclinical models of pancreatitis have shown increased sensitivity of the pancreas using electrical stimulation, as well as dermal hypersensitivity (detection of a stimulus not normally detectable) in regions both local (abdomen) and distant (hindpaw) to the site of inflammatory origin [43–45, 61], suggesting activation of both normal pain pathways and central sensitization. While alterations to peripheral Aδ and C fiber activity promote the induction of pain [62, 63], peripheral targets such as ion channels regulating neural sensitization [64, 65], and release of chemical mediators of inflammation such as pro-inflammatory cytokines e.g. TNF/TNFR1 also modulate these effects [64, 66–68], and can occur at the level of the nociceptors [69, 70], dorsal root ganglion [67, 71], thalamus [72], and somatosensory cortex [72]. Central plasticity also overlays these pain pathways, involving the change of connectivity between central brain structures, leading to loss of input to cortical pain sensing regions (e.g. somatosensory and prefrontal cortices) [73, 74]. Strong evidence also supports of role of TNF throughout the limbic system to regulate pain [72, 75, 76], perhaps by incorporating emotional memories of pain. One region of the limbic system that has garnered much attention is the hippocampus whereby hippocampal TNF/TNFR1 activity regulates the severity of neuropathic pain [75–78]. Indeed, one of our important findings in these studies is the improvement of neuropathic pain over the course of the study in the XPro1595-treated mice, even after the resolution of inflammation in all groups by day 7. This suggests that the early induction of inflammation in these mice is a significant contributor to the persistence of pancreatitis-induced pain, which we have shown can be alleviated by selectively inhibiting solTNF. Our data also shows a strong tendency for upregulation of GFAP in the hippocampus a week after the induction of acute pancreatitis, despite the resolution of pancreatic inflammatory cells at this timepoint. This upregulation supports the notion that systemic inflammation may persist despite local resolution. Since astrocytes are known to contribute to excess levels of TNF [79], and hippocampal TNF/TNFR1 activity is known to promote neuropathic pain [76, 78], it is plausible to suggest that hippocampal TNF/TNFR1 activity could be contributing to the induction of neuropathic pain in our model of acute pancreatitis. However, the subcutaneous administration of XPro1595, while clinically relevant, prevents determination of the molecular mechanisms involved. None-the-less, the improvement in pancreatic pathology and associated pain, combined with a lack of known side-effects in both animal models and patients, supports the use of XPro1595 clinically in patients experiencing acute pancreatitis.

## Conclusion

Excess levels of the inflammatory cytokine TNF plays a prominent role in many inflammatory disease pathologies, including the induction of pancreatitis. Attempts to use TNF receptor fusion proteins or monoclonal antibodies to regulate this cytokines function have shown some successes clinically, but has been fraught with complications due to their numerous adverse side-effects, including drug-induced acute pancreatitis. Our data provide support for the clinical use of a novel “second generation” TNF inhibitor XPro1595 that selectively inhibits only the detrimental soluble form of TNF to prevent the disease sequelae, while sparing the beneficial transmembrane form of TNF to allow reparative cellular mechanisms to remain.

## Data Availability

The datasets generated and/or analyzed during the current study are available from the corresponding author on reasonable request.

## Abbreviations

LTP: Long term potentiation
solTNF: Soluble form of tumor necrosis factor
tmTNF: Transmembrane form of tumor necrosis factor
TNFR1: Tumor necrosis factor receptor 1
TNFR2: Tumor necrosis factor receptor 2
